# The papers presented at 7th Young Scientists School “Systems Biology and Bioinformatics” (SBB’15): Introductory Note

**DOI:** 10.1186/s12863-015-0326-5

**Published:** 2016-01-27

**Authors:** Ancha V. Baranova, Yuriy L. Orlov

**Affiliations:** Research Center for Medical Genetics RAMS, Moscow, Russian Federation; Center for the Study of Chronic Metabolic Diseases, School of System Biology, George Mason University, Fairfax, VA USA; Institute of Cytology and Genetics, Siberian Branch, Russian Academy of Sciences, Lavrentieva ave., 10, Novosibirsk, 630090 Russian Federation; Novosibirsk State University, Pirogova, 2, Novosibirsk, 630090 Russian Federation

A selection of papers prepared by participants of 7th Young Scientists School SBB’15 is presented in three Supplementary Issues to the journals of BMC series, namely, BMC Genomics, BMC Genetics and BMC Microbiology.

SBB’15 took place June 22nd-25th in Novosibirsk, Russia (http://conf.nsc.ru/sbb2015). The series of Young Scientists Schools in Systems Biology and Bioinformatics (SBB) started several years ago, in 2008, as educational workshop associated with the large international conference series on bioinformatics known as BGRS\SB (Bioinformatics of Genome Regulation and Structure\Systems Biology) (http://conf.bionet.nsc.ru/bgrssb2016). After its inauguration year of 1998, BGRS/SBB held biannually at the Institute of Cytology and Genetics SB RAS in Novosibirsk. In a recent decade, it became a prime meeting venue for biologists, computer scientists, physicists, mathematicians and biochemists working in an interdisciplinary field of systems biology and computer genomics. Being the largest system biology meeting series in Russia, BGRS\SB-14 attracted participants from 27 countries. In the past BioMedCentral had published series of special issues based on best materials presented at the conference in BMC Genomics (http://www.biomedcentral.com/bmcgenomics/supplements/15/S12), BMC Evolutionary Biology (http://www.biomedcentral.com/bmcevolbiol/supplements/15/S1), BMC Genetics (http://www.biomedcentral.com/bmcgenet/supplements/16/S1) and BMC Systems biology (http://www.biomedcentral.com/bmcsystbiol/supplements/9/S2). In addition, the protein structure related studies presented by BGRS/SBB participants were collated as special issues in Journal of Bioinformatics and Computational Biology (http://www.worldscientific.com/toc/jbcb/4113/01; http://www.worldscientific.com/toc/jbcb/11/01). [1]

In addition, the SBB School series has its own history to be proud of. The first generation of its participants already matured enough to substantially advance in the field. A majority of the participants graduated with PhDs, and now are contributing to the school as the lecturers and to a number of world universities as professors. For some students, the conference became instrumental in obtaining their post-doc or first independent position in academic world. In many cases, the informal atmosphere of the school helped to spark a collaboration that later led to publications in leading journals or the dramatic changes in their science career.

The Program Committee of the conference and SBB Schools series includes data analysis, bioinformatics and computational biology professionals from different countries. For the last decade, the SBB conference was co-chaired by Academician Prof. Nikolay A. Kolchanov of the Institute of Cytology and Genetics SB RAS, Russia and Prof. Dr. Ralf Hofestädt of Bielefeld University, Germany.

System biology and bioinformatics are rapidly developing and wide fields of science knowledge. For Young Scientists Schools, every year is different as it comprises the topics for the trainings that are the hottest at the time of the event. In 2015, the SBB School concentrated on the modern genomics and an analysis of high-throughput sequencing data. The materials presented in the current special issue provide examples of seamless integration of experimental studies performed in the lab and the computer-assisted inquiries into in the complex patterns of the organization and the functioning of biologic systems at the molecular, cell, tissue, organ, and body levels.

The scientific topics discussed in 2015 were presented at the following sections:

1. Next generation sequencing (NGS) and data analysis
2. Evolutionary bioinformatics
3. Systems biology and gene network modeling

In SBB’15 Supplements to BMC journals Genomics, we collected the best studies presented at the conference. Due to the general breadth of the field of Systems Biology and Bioinformatic, the manuscripts were accepted into three separate tracks, thus forming three Supplementary Issues, BMC Genomics, BMC Genetics and BMC Microbiology. Below we will describe contents of these issues.

## BMC genetics

The paper by Fedoseeva et al. titled “Comparative transcriptional profiling of renal cortex in rats with Inherited Stress-Induced Arterial Hypertension and normotensive Wistar Albino Glaxo rats” dissect genetic underpinnings of arterial hypertension, a disease which cannot be explained as progression along a straightforward route [2]. Hence, the entire biochemical network that runs within the renal cortex was considered, with every participating gene classified as either an element in the process imbalanced or otherwise disturbed due to the development of pathology, or as the compensatory change evoked in the processes of an attempt to restore the homeostasis.

The paper of Klimov et al. “Genome-wide transcriptome analysis of hypothalamus in rats with inherited stress-induced arterial hypertension” continues in the wake of previous paper with a systems biology analysis of expression changes in hypothalamus of the same two strains of rats, and pinpointed the changes within three categories of genes: ‘the regulation of hormone level’, ‘the regulation of the blood pressure level’, and ‘the immune system processes’ [3].

The paper of Lavrov and co-authors titled “Frequent variations in cancer-related genes may play prognostic role in treatment of patients with chronic myeloid leukemia” convincingly demonstrated that the gene variants present or absent in the genome of patient influence relative efficiency of the therapy for chronic myeloid leukemia, thus, paving a way to personalized approach to the treatment of this disease [4].

Finally the paper “Genomic Determinants of Birth Weight Variability in the Pig” by Wang and co-author explored the polimorphisms influencing the weight of newborn piglets, and highlighted a number of genes involved in glucose and lipid metabolism as well as in maternal-fetal lipid transport as important for this agriculturally important trait. Additionally, there a basic science related vibe in this study as it may provide some mechanistic explanation to relative success of intrauterine competition of the fetuses in species with larger size of litter [5].

## BMC genomics

The paper by Ivanov et al.“Non-random fragmentation patterns in circulating cell-free DNA reflect epigenetic regulation” [6] convincingly demonstrates that the patterns of the sequenced fragments’ ends distribution in cell free fDNA to some degree reflect the epigenetic markings in its tissue of origin. The implications of this study are multiple. If, indeed, the cfDNA patterning reflects a general picture of gene expression, then the mapping and the mining of cfDNA fragment ends may yield of novel pathologically relevant biomarkers reflecting pathological changes in chromatin marks. Moreover, the obvious fragment ends patterning observed both in genomic DNA and in cfDNA may allow fine tuning the primer positions to achieve higher amplification yields in PCR detection of point mutations in formalin fixed samples that are well known for relative difficulty of PCR-based mutation detection. (http://www.biomedcentral.com/1471-2164/16/S13/S1).

The paper by Medvedeva et al. “Computer analysis of protein functional sites projection on exon structure of genes in Metazoa” [7] (http://www.biomedcentral.com/1471-2164/16/S13/S2) deals with the peculiarities of the exon-intron structure, which affect the functionality of the encoded protein. The paper shows that protein functional sites predominantly encoded by relatively long exons.

The paper by Petrovskiy et al. “Prediction of tissue-specific effects of gene knockout on apoptosis in different anatomical structures of human brain” (http://www.biomedcentral.com/1471-2164/16/S13/S3) describes a theoretical model that assesses tissue-specific gene knockout effect on gene expression in various structures of the brain, and predicts that the most pronounced tissue-specific effects are detected for genes that participate in the apoptosis/survival network [8].

Gunbin and colleagues continued the evolutionary analysis of miRNA sequences in brain using unique paleogenetics data described in the manuscript titled “The evolution of *Homo sapiens denisova* and *Homo sapiens neanderthalensis* miRNA targeting genes in the prenatal and postnatal brain” [9] (http://www.biomedcentral.com/1471-2164/16/S13/S4).

The paper by Arkova et al. “Obesity-related known and candidate SNP markers can significantly change affinity of TATA-binding protein for human gene promoters” [10] (http://www.biomedcentral.com/1471-2164/16/S13/S5) presents an *in silico* analysis of 22 known nucleotide polymorphisms and arrived at functionally important conclusion about the mechanism of action for at least one of these DNA variants.

Finally, the works by Menzorov et al. [11], Kozlov et al. [12] and Moskalev et al. [13] describe the transcriptome analysis of American mink (Menzorov et al. “Comparison of American mink embryonic stem and induced pluripotent stem cell transcriptomes” http://www.biomedcentral.com/1471-2164/16/S13/S6), the functional transcription factor binding sites ranking in the fruitflies (Kozlov et al. “Analysis of functional importance of binding sites in the *Drosophila* gap gene network model” (http://www.biomedcentral.com/1471-2164/16/S13/S7), and comparative mining of stress- related expression profiles (Moskalev et al. “A comparison of the transcriptome of *Drosophila melanogaster* in response to entomopathogenic fungus, ionizing radiation, starvation and cold shock” (http://www.biomedcentral.com/1471-2164/16/S13/S8).

## BMC microbiology

The paper by Klimenko et al. titled “Bacteriophages affect evolution of bacterial communities in spatially distributed habitats: a simulation study” explored evolutionary scenarios that may be influenced by the spatial location of initial phage invasion and showed that the speciation rate is lower when invasion penetrated the fully formed community of sedentary cells [14].

In their paper “On the control mechanisms of the nitrite levels in Escherichia coli cells” Khlebodarova, Ree and Likhoshvai describe the mathematical model of nitrite utilization in E.coli cells cultured in the flow chemostat, the process relevant to many biotechnological and clinical applications [15].

The paper of Bryanskaya et al. titled “The role of environmental factors for the composition of microbial communities of saline lakes in the Novosibirsk region (Russia)” for the first time described microbial composition of these saline lakes, their dependence on physical-chemical parameters of waters, as well as evaluated possible avenues for their bioprospecting [16].

We wish you an interesting reading and sincerely await you at our next BGRS conference that will take place in Novosibirsk 29 August – 2 September 2016 (http://conf.bionet.nsc.ru/bgrssb2016/).
